# Successful long-term remission through tapering tocilizumab infusions: a single-center prospective study

**DOI:** 10.1186/s41927-019-0109-0

**Published:** 2020-02-28

**Authors:** Chayma Ladhari, Pierre Le Blay, Thierry Vincent, Ahmed Larbi, Emma Rubenstein, Rosanna Ferreira Lopez, Christian Jorgensen, Yves-Marie Pers

**Affiliations:** 1 IRMB, University of Montpellier, Inserm U1183, CHU Montpellier, 371, avenue du doyen Gaston Giraud, 34295 Montpellier, France; 2 Department of Immunology, Saint Eloi University Hospital, 80 rue Augustin Fliche, 34295 Montpellier Cedex 5, France; 3 Department of Radiology, CHU Nimes, Place du Pr R. Debré, 30029 Nîmes Cedex 9, France

**Keywords:** Rheumatoid arthritis, Tocilizumab, Maintenance, Tapering, Remission

## Abstract

**Background:**

Strategic drug therapy for rheumatoid arthritis (RA) patients with prolonged remission is not well defined. According to recent guidelines, tapering biological Disease-Modifying Anti-Rheumatic Drugs (bDMARDs) may be considered. We aimed to evaluate the effectiveness of long-term maintenance of tocilizumab (TCZ) treatment after the progressive tapering of infusions.

**Methods:**

We conducted an exploratory, prospective, single-center, open-label study, on RA patients with sustained remission of at least 3 months and treated with TCZ infusions every 4 weeks. The initial re-treatment interval was extended to 6 weeks for the first 3 months. Thereafter, the spacing between infusions was determined by the clinician. Successful long-term maintenance following the tapering of TCZ infusions was defined by patients still treated after two years by TCZ with a minimum dosing interval of 5 weeks.

**Results:**

Thirteen patients were enrolled in the study**.** Eight out of thirteen were still treated by TCZ after two years**.** Successful long-term maintenance was possible in six patients, with four patients maintaining a re-treatment interval of 8-weeks or more. We observed 5 patients with TCZ withdrawal: one showing adverse drug reaction (neutropenia) and four with secondary failure. Patients achieving successful long-term maintenance with TCZ were significantly younger than those with secondary failure (*p* < 0.05). In addition, RA patients with positive rheumatoid factor and anti-citrullinated peptide antibodies, experienced a significantly greater number of flares during our 2-year follow-up (*p* < 0.01).

**Conclusions:**

A progressive tapering of TCZ infusions may be possible for many patients. However, larger studies, including more patients, are needed to confirm this therapeutic option.

**Trial registration:**

NCT02909998. Date of registration: October 2008.

## Background

Rheumatoid arthritis (RA) is the most common inflammatory rheumatic disease. New licensed biological agents are now commonly used in RA treatment. Recent recommendations from the American College of Rheumatology (ACR) and European League Against Rheumatism (EULAR) highlighted the need to treat RA patients quickly in order to obtain clinical remission without radiological damage [1, 2]. This therapeutic goal aims to prevent structural damage and disease progression. Clinical remission is defined by the absence of symptoms of disease activity and numerous validated indexes are able to classify patients (DAS28 < 2.6 or CDAI < 2.8; SDAI < 3.3 or Boolean criteria) [3, 4]. The new EULAR guidelines stress the importance of a “treat-to-target” strategy and outline recommended changes in therapy for patients exhibiting disease remission. In the context of persistent remission (up to 6 months), ending steroid treatment as quickly as possible is recommended [5]. Then, if the patient remains in remission, clinicians can consider tapering biological disease-modifying anti-rheumatic drugs (bDMARDs), either through reduction of dose or extension of the interval between applications (‘spacing’) [5]. Furthermore, the updated EULAR recommendations for the management of RA with synthetic and bDMARDs highlight early RA, the depth of improvement, and the duration of remission as predictors of the likely success of tapering [5].

Increasing the spacing between biological agents, or stopping them altogether may also be desirable for safety reasons as well as issues of health economics [6]. As biologics are more expensive than conventional synthetic (cs) DMARDs, and may cause more serious adverse events, the next step in RA therapy management should be to assess the possibility of sustaining remission without the use of biologics. Some studies of tumor necrosis factor α (TNF-α)-targeting drugs found that dose reduction or discontinuation of biological agents can be achieved in a relevant proportion of RA patients without loss of disease control or radiological damage [7–9]. Indeed, more than one-third of RA patients with low disease activity (LDA) or in remission did not experience a disease flare within the first year of tapering or stopping DMARD treatment [10].

Tocilizumab (TCZ) is the first therapeutic agent targeting IL-6 to be effective in RA treatment. TCZ is approved for the treatment of active, moderate-to-severe RA in patients who have had an inadequate response to one or more csDMARDs and/or TNF antagonists [11]. Recent studies suggest that TCZ could induce drug-free remission in a low proportion of patients [12, 13]. However, few data reporting the efficacy and safety of TCZ tapering are available and the follow-up of patients is short (less than one year) [14]. We hypothesized that increasing the interval or spacing between TCZ infusions, rather than abruptly stopping treatment in RA patients with remission, may represent a better strategy to reduce the risk of flare. We assumed that the efficacy of TCZ dose reduction may not be equivalent to a dosing interval extension for pharmacokinetics/pharmacodynamics reasons [15]. However, we chose the spacing strategy because it was more convenient and less costly in “real life” practice due to reducing the hospital stay. Thus, our aim was to evaluate the long-term maintenance of remission after progressive TCZ tapering in RA patients.

## Methods

### Patients’ characteristics

We conducted a prospective, exploratory, single-center, open label study with RA patients fulfilling the 2010 ACR/EULAR criteria (NCT02909998-ClinicalTrials.gov). We included patients in remission for at least 3 months and treated by TCZ infusions every 4 weeks. Criteria for remission were defined by DAS28 < 2.6 with only one swollen joint authorized in a 44-joint swollen count. The RA patients were without corticosteroids for at least 4 weeks and with a stable dose of csDMARDs for at least 3 months.

The study was approved by the local ethics committee (CPP IV Sud-Méditerranée, Montpellier, France) in accordance with the Helsinki Declaration and informed consent was obtained from each patient (NCT02909998; N°2008-A01087-48). Patients were recruited when they reported for their regular monthly infusion.

### Design

The initial assessment included clinical, biological, and Doppler ultrasound (US) parameters for hands and feet. Doppler US was performed at baseline for all patients by the same radiologist with experience in rheumatic and musculoskeletal diseases. Sera of the patients was collected the day prior to the start of spacing to define levels of plasma TCZ and antidrug antibodies (ADAb). Samples were analyzed in the department of Immunology using LISA-TRACKER® enzyme-linked immunosorbent assay (ELISA) kits (Theradiag, Marne La Vallée, France).

Retreatment interval (RTI) was fixed at 6 weeks spacing for the first 3 months. Thereafter, if possible, TCZ tapering to 8 weeks was advised, although the exact spacing pattern was left to be determined by the clinician. A flare could occur during the prolongation of TCZ administration interval and was defined by a DAS28 > 2.6 with a progression in the DAS28 > 0.6 compared to previous DAS28 [3]. In the case of flare, patients could be returned to a 4 or 6-weeks interval. Other options for clinicians were also available, such as maintaining the RTI through decreasing TCZ doses, optimizing csDMARDs or introducing corticosteroids.

### Outcome assessment

Successful long-term maintenance after tapering was defined after 2 years by the percentage of patients still treated by TCZ and with at least a 5-week interval between two infusions. The rate and time to flare after progressive TCZ spacing and predictors of maintaining remission or flare after tapering were analyzed. Possible reasons for TCZ withdrawal or spacing failure were also analyzed.

### Statistical analysis

A general description of the sample was performed using the frequencies for qualitative variables and the mean, standard deviation and range are reported for quantitative variables. Comparisons between groups of patients were performed using a Wilcoxon-Mann-Whitney test for averages and a Fisher’s exact test for contingency tables of qualitative variables. The significance level was set at 5% for all tests. Statistical analyses were performed using Prism version 6.0c (GraphPad Software Inc., La Jolla, CA, USA).

## Results

### Characteristics of patients

From July 2011 to September 2012, thirteen RA patients who met the inclusion criteria were recruited into the study. Baseline patient characteristics are summarized in Table 1. Most patients were female (69.2%) with a mean age of 48.2 ± 14.5 years. The mean disease duration was 11.5 ± 9.4 years. Mean TCZ-treatment duration prior to initiating spacing was 18.4 ± 7.3 months. The mean time of remission was 7.5 ± 6.2 months. Ten patients (76.9%) presented erosions at baseline and seven (53.8%) were positive for rheumatoid factor (RF) and anti-citrullinated peptide antibodies (ACPA). Prior to TCZ treatment, the mean number of previous csDMARDs and bDMARDs used were 2.1 ± 0.9 and 1.7 ± 1.2, respectively. None of the patients in the study had received rituximab prior to TCZ treatment. Six patients received concomitant treatment with methotrexate (MTX), three with leflunomide, and five received TCZ in monotherapy. At the start of the progressive spacing, the mean DAS28 score was 1.6 ± 0.9.

**Table 1:** Baseline RA patient’ characteristics

	All patients (*n* = 13)
Age, mean (SD), years; (min-max)	48.23 ± 14.5, (21–67)
Female, n (%)	9 (69.2%)
Disease duration, mean (SD), years; (min-max)	11.46 ± 9.44; (2–36)
TCZ exposure before tapering, months (SD); (min-max)	18.38 ± 7.34; (5–29)
Remission achievement delay, months (SD); (min-max)	2.38 ± 2.63; (0–8)
Remission duration, months (SD); (min-max)	7.53 ± 6.21; (3–24)
RF positive, n (%)	7 (53.8%)
ACPA positive, n (%)	7 (53.8%)
Erosive status, n (%)	10 (76.9%)
Previous number of sDMARDs; (min-max)	2.15 ± 0.9; (1–4)
Previous number of bDMARDs; (min-max)	1.7 ± 1.18; (0–3)
Swollen joint count; (min-max)	0.08 ± 0.27; (0–1)
Tender joint count; (min-max)	3.14 ± 3.4; (0–10)
Hyperhemia at Doppler	2 (15.4%)
Number of patients with active synovitis	7 (53.8%)
Number of active synovitis	
Hands	14
Feet	5
Concomitant therapy	
MTX, n (%)	6 (42.9%)
LEF, n (%)	3 (21.4%)
DAS28; mean (SD); (min-max)	1.65 ± 0.93; (0–2.6)
ESR, mm/h; mean (SD); (min-max)	7 ± 9.12; (1–27)
CRP, mg/L, mean (SD); (min-max)	2.21 ± 2.34; (0.2–7.4)
TCZ plasma level, mg/L; mean (SD); (min-max)	7.35 ± 6.41; (0–16)
ADAb positivity	0

*RA* rheumatic arthritis, *SD* standard deviation, *n* number, *TCZ* tocilizumab, *RF* rheumatoid factor, *ACPA* anti-citrullinated peptide antibodies, *MTX* methotrexate, *LEF* leflunomide, *DAS28* Disease Activity Score in 28 joints, *ESR* erythrocyte sedimentation rate, *CRP* C-reactive protein, *ADAb* antidrug antibodies

### Patient outcomes after tapering TCZ infusions

After a 2-year follow-up, 8/13 patients remained on TCZ therapy after the spacing attempt. Successful tapering of TCZ treatment with a long-term controlled disease and a minimum 5-week interval between infusions, was achieved for six patients (46.1%) (Table 2). Among these patients, four were maintained on a RTI of eight or more weeks, and their mean DAS28 score at 24 months was 1.58 ± 0.6.

**Table 2:** Evolution of RA patients’ disease activity during the 24-months follow-up

		Day 0	W6	M3	M6	M9	M12	M18	M24
P1	DAS 28	1.76	**3.14**	2.43	2.43	1.76	1.55	**5.18**	1.64
	RTI	4	6	6	6	6	8	8	6
	Dose	4	4	4	4	4	4	4	4
P2	DAS 28	1.69	2.35	2.79	**2.89**	2.69	**3.14**	1.34	2.13
	RTI	4	6	6	8	6	8	4	4
	Dose	8	8	8	8	8	8	8	8
P3	DAS 28	2.4	2.07	2.57	**3.92**	2.14	**3.7**	**3.68**	2.61
	RTI	4	6	6	8	4	4	5	4
	Dose	8	8	8	8	8	8	8	8
P4	DAS 28	2.6	1.42	1.34	1.94	1.05	1.12	**3.35**	1.49
	RTI	4	6	8	8	4	5	5	5
	Dose	8	8	8	8	8	8	8	8
P5	DAS 28	2.58	2.6	1.61	2.11	2.23	2.22	2.03	0.91
	RTI	4	6	6	7	8	8	9	9
	Dose	8	8	8	8	8	8	8	8
P6	DAS 28	0.48	NA	0.76	1.37	0.28	0.28	2.22	1.84
	RTI	4	6	7	6	6	6	8	9
	Dose	8	8	8	8	8	8	8	8
P 7	DAS 28	1.61	NA	2.57	1.34	1.94	**3.28**	2	2.5
	RTI	4	5	8	8	8	8	8	8
	Dose	6	8	8	6	6	6	8	8
P8	DAS 28	1.98	2.26	1.68	2.14	NA	1.81	2.42	Secondary failure Switch to infliximab
	RTI	4	6	6	8	8	8	4	
	Dose	8	8	8	8	8	8	4	
P9	DAS 28	0.07	1.45	1.89	1.89	1.12	0.76	0.76	1.12
	RTI	4	6	6	7	7	8	9	10
	Dose	8	8	8	8	8	8	8	8
P10	DAS 28	1.38	**3.2**	NA	2.85	2.45	3.71	Secondary failure Switch to abatacept	
	RTI	4	6	4	4	4	4		
	Dose	8	8	8	8	8	8		
P11	DAS 28	2.31	2.31	**3.48**	0.85	Neutropenia Switch to abatacept			
	RTI	4	5	6	4				
	Dose	4	4	6	6				
P12	DAS 28	2.56	1.03	**4.08**	**3.51**	Secondary failure Switch to abatacept			
	RTI	4	6	6	4				
	Dose	8	8	8	8				
P13	DAS 28	0	1.03	1.55	2.57	**5.35**	Secondary failure Switch to abatacept		
	RTI	4	6	6	8	6			
	Dose	6	6	6	6	6			

*Dose* mg/kg, *W* week, *M* month, *P* patient, *RTI* retreatment interval, *NA* not available

The successful long-term maintenance group (6/13) experienced on average one flare ±0.9 during the study, with a mean delay of occurrence of 4.4 ± 4.9 months after the start of spacing. Only two patients remained on a 4-week RTI of TCZ infusions. A switch to another biologic was needed for five patients, four of which experienced a secondary failure (one was switched to anti-TNF-α and the other three to abatacept). The remaining patient developed a severe TCZ-induced neutropenia.

### Predictors of maintaining remission or flare after tapering

In order to evaluate potential predictors of maintaining remission following TCZ tapering, we compared patients experiencing secondary failure (*n* = 4) with those experiencing successful long-term maintenance (*n* = 6) (Table 3). Patients with secondary failure were significantly older than patients experiencing successful long-term maintenance (mean age: 60.7 ± 6.7 and 41.8 ± 15.2 respectively; *p* = 0.038). Hand and feet US characteristics were similar in each group. TCZ plasma level was similar in both groups and none of them developed ADAb. There was a marginally non-significant tendency (*p* = 0.07) for patients in the successful long-term maintenance group to experience fewer flares during the 2 years of the study (mean number of flares: 2 ± 0 versus 1 ± 0.9).

**Table 3:** Comparison of baseline characteristics between successful long-term maintenance group (*n* = 6) vs. Secondary failure group (*n* = 4)

	Secondary failure (*n* = 4)	Patients with successful long-term maintenance (*n* = 6)	*p*-value
Age, mean (SD), years	60.75 ± 6.7	41.8 ± 15.23	*<* 0.05
Female, n (%)	2 (50%)	4 (66.7%)	NS
Disease duration, mean (SD), years	15 ± 14.07	7.8 ± 4.35	NS
TCZ exposure before tapering, months (SD)	19.5 ± 7.23	14.8 ± 7.5	NS
Remission achievement delay, months (SD)	3.5 ± 3.4	2.16 ± 2.4	NS
Remission duration, months (SD)	8 ± 5.7	4.8 ± 2.13	NS
RF positive, n (%)	3 (75%)	2 (33.3%)	NS
ACPA positive, n (%)	3 (75%)	2 (33.3%)	NS
Erosive status, n (%)	4 (100%)	4 (66.7%)	NS
Previous number of sDMARDs	2.75 ± 1.25	2 ± 0.63	NS
Previous number of bDMARDs	1.5 ± 1.73	1.66 ± 1.21	NS
Swollen joint count	0	0.166 ± 0.4	NS
Tender joint count	2.25 ± 2.6	3.16 ± 4.57	NS
Hyperhemia at Doppler	1 (25%)	1 (16.7%)	NS
Number of patients with active synovitis	1 (25%)	4 (66.7%)	NS
Number of active synovitis			
Hands	8	4	NS
Feet	0	5	NS
Concomitant therapy			
MTX, n (%)	2(50%)	2(33.3%)	NS
LEF, n (%)	0(0%)	2 (33.3%)	NS
DAS28; mean (SD)	1.48 ± 1.1	1.51 ± 1.05	NS
ESR, mm/h; mean (SD)	7.5 ± 11	6.66 ± 10.1	NS
CRP, mg/L, mean (SD)	2.55 ± 3.3	3 ± 1.87	NS
TCZ plasma level, mg/L; mean (SD)	7.12 ± 7.7	7.35 ± 6.41	NS
ADAb positivity	0	0	NS
Flare, mean (SD)	2 ± 0	1 ± 0.9	0.07

*SD* standard deviation, *n* number, *TCZ* tocilizumab, *RF* rheumatoid factor, *ACPA* anti-citrullinated peptide antibodies, *MTX* methotrexate, *LEF* leflunomide, *DAS28* Disease Activity Score in 28 joints, *ESR* erythrocyte sedimentation rate, *CRP* C-reactive protein, *ADAb* antidrug antibodies, *NS* non significant

Lastly, we compared patients who experienced one flare or less during the whole study with the remaining patients who experienced two or more (Table 4). While none of the baseline clinical, biological, and imaging characteristics were associated with successful tapering of TCZ infusions, we found that RF and ACPA positivity were both associated with a greater number of flares (*p* = 0.004). A multivariate analysis found no significant independent predictors of maintaining remission or flare occurrence after tapering.

**Table 4:** Comparison between patients with one flare or less vs. patients with more than a flare

	Patients with one flare or less (*n* = 5)	Patients with more than a flare (*n* = 8)	p-value
Age, mean (SD), years	40.8 ± 15.7	52.9 ± 12.5	NS
Female, n (%)	3 (60%)	6 (75%)	NS
Disease duration, mean (SD), years	12.4 ± 8.7	10.9 ± 10.4	NS
TCZ exposure before tapering, months (SD)	16.4 ± 8.8	20.4 ± 5.7	NS
Remission achievement delay, months (SD)	3 ± 2.4	2 ± 2.8	NS
Remission duration, months (SD)	5.6 ± 2.8	8.8 ± 7.5	NS
RF positive, n (%)	0 (0%)	7 (87.5%)	0.004
ACPA positive, n (%)	0 (0%)	7 (87.5%)	0.004
Erosive status, n (%)	3 (60%)	7(87.5%)	NS
Previous number of sDMARDs	1.8 ± 0.8	2.4 ± 1	NS
Previous number of bDMARDs	1.6 ± 1.1	1.75 ± 1.3	NS
Swollen joint count	0	0.125 ± 0.3	NS
Tender joint count	2.4 ± 4.3	3.6 ± 2.97	NS
Hyperhemia at Doppler	0 (0%)	(37.5%)	NS
Number of patients with active synovitis	3 (60%)	4(50%)	NS
Number of active synovitis			
Hands	3	11	NS
Feet	2	3	NS
Concomitant therapy			
MTX, n (%)	3 (60%)	3 (37.5%)	NS
LEF, n (%)	1 (20%)	2 (25%)	NS
Flare, mean (SD)	0.6 ± 0.5	2.13 ± 0.35	0.001

*SD* standard deviation, *n* number, *TCZ* tocilizumab, *RF* rheumatoid factor, *ACPA* anti-citrullinated peptide antibodies, *MTX* methotrexate, *LEF* leflunomide, *NS* non-significant

## Discussion

Our observation of sustained remission in eight of our thirteen patients suggests that maintenance of TCZ therapy may be feasible following attempts to increase the spacing of infusions. Indeed, six patients successfully transitioned to long-term maintenance with tapered TCZ infusions. Four patients developed a secondary failure after beginning the spacing of infusions, while one patient developed severe neutropenia associated with TCZ. The age of patients may influence the success of long-term maintenance on TCZ, as younger patients were more likely to experience a successful transition and less likely to experience secondary failure. Moreover, RF and ACPA positive RA patients experienced more episodes of flares during our follow-up, underscoring a greater severity of the disease.

Guidelines concerning initiation of bDMARDs and how to induce remission are well established [2, 16]. However, data on patient responses to therapy once remission is reached are scarce. Stopping bDMARDs after achieving remission is challenging due to a potential tradeoff between the important health economic impact that could be achieved on one hand and the potential risk of recurrence on the other [17]. New EULAR recommendations propose that clinicians consider changes in therapy, either through changes in dose or increasing the spacing between treatments, especially for patients in long-term remission in association with csDMARDs [5]. However, recommended strategies are not yet clearly defined and the consequences of such changes are not well understood. Cost-analysis studies clearly demonstrate that decreasing doses of bDMARDs decreases costs [18]. What remains unclear are the consequences for patients, both in terms of identifying the long-term consequences of extending dosing (radiographic changes, flares ...) as well as determining characteristics that may aid clinicians in identifying patients in which down-titration or discontinuation of bDMARDs may be possible [18].

Several studies have examined the discontinuation or changing the spacing of bDMARDs in RA patients with prolonged remission. Results suggest that discontinuation of anti-TNF-α agents does not allow a sustained remission in most drug-free patients [7, 19]. Indeed, the prospective observational study of Van der Mass et al. found that discontinuation of anti-TNF-α therapy was feasible for only 16% of their patients [7]. The PRESERVE study compared three strategies of RA drug management (maintenance, half dose reduction and complete discontinuation of etanercept) in a randomized controlled trial of RA patients showing sustained remission in the past year [19]. After 1 year, low disease activity (LDA) was observed in 46% of patients in the placebo group versus 82.6% in patients maintained on etanercept therapy. In addition, the ADMIRE study with adalimumab [20], CERTAIN study with certolizumab pegol [21] and DOSERA study with etanercept [22] all demonstrated that withdrawing anti-TNF-α therapy did not allow maintenance of patients in sustained remission, with rates of sustained remission of 13, 17 and 13% respectively. In light of these results, others have tried down-titration of bDMARDs, rather than discontinuation, to maintain RA patients in sustained remission. In the PRESERVE study, Smolen et al. found that the risk of relapse and structural damage progression in patients switched to a half dose was statistically indistinguishable from those maintained with a full dose after one year [19]. The recent STRASS study of RA-patients in remission (DAS28 < 2.6) found that relapse was observed more frequently in patients placed on a progressively increased spacing of TNF-blockers injections than those maintained on their previous dosing regimen (76.6% vs 46.5%, *p* = 0.0004), however the equivalence of the two strategies could not be demonstrated due to an underpowered trial [23]. Most recently, the TARA (TApering strategies in Rheumatoid Arthritis) study, the first randomized controlled study comparing a de-escalation strategy with reduction and discontinuation of synthetic DMARDs versus TNF-blockers, highlighted the importance of first reducing treatment with bDMARDs prior to initiation of synthetic DMARDs therapy [24].

Concerning TCZ tapering, several strategies have been tried, including discontinuation TCZ infusions [12, 13], and gradually increasing the RTI to a fixed interval (eg. 6-weeks) [14, 25–27]. We conducted the present study to determine if tapering TCZ by progressively increasing the spacing of infusions would be a better strategy to maintain TCZ-treated patients in remission. This approach was adopted because we believed that it might be the best therapeutic option for our patients as well as from a medico-economical standpoint. A recent study extending the dosing interval of TCZ infusions to 6 weeks for RA patients in sustained remission for the previous 3 months appeared to provide an acceptable option, with 88% retaining remission after 54 weeks [14]. Similarly, Saiki et al. published two studies on extending the RTI for TCZ infusions. In the first, they showed the feasibility of 5-week or 6-week spacing in patients with low disease activity, with a therapeutic maintenance rate of 90% at two years [25]. In the second study, 60% of patients who passed directly to a treatment interval of 6 weeks remained on treatment two years later [26]. In another Dutch study, testing the strategy first reducing infusion doses to 4 mg/kg and then progressively increasing the spacing between treatments, 42% of patients responded positively to TCZ de-escalation at one year [27]. By contrast, sustained remission following cessation of TCZ treatment was much lower: in the DREAM study [13], drug free remission or LDA was present in 35.1, and 13.4% of patients after 24 and 52 weeks respectively following cessation of TCZ used in monotherapy, and in the ACT-RAY study, the remission rate dropped to 16% after stopping TCZ [28]. In our study, we observed that a majority (61.5%) of our patients remained in remission at month 24 and 46.1% had successful long-term maintenance following the tapering of TCZ infusions, results that are comparable to those of previous studies [14, 25, 26].

Some factors contributing to prolonging the duration of DAS28 remission and LDA after discontinuation or tapering of biologics have been previously identified. A lack of disease severity factors may act as selection criteria for informing decisions about whether or not to increase spacing of TCZ infusions [13]. In the BeST study and in early arthritis cohorts, successful discontinuation was associated with the absence of ACPA, male gender, rapid achievement of remission, non-smoking and absence of an HLA shared epitope [9, 29]. A shorter disease duration, better functional ability at discontinuation, and shorter symptom duration before starting any treatment were also predictive of successful discontinuation of bDMARDs [17]. Our analysis of various clinical, radiological, biological and immune variables (Plasma TCZ level and ADAb), identified only the absence of RF and ACPA as being significantly associated with fewer flares.

Our study is mainly limited by the small number of included patients. As such, this preliminary analysis represents a “proof of concept”. In addition, our cohort was composed of patients with very severe disease (more than 75% with erosions, and long disease duration), none of whom had started TCZ as a first line of bDMARD. We also chose a very strict definition of remission in order to reduce the risk of relapse. The risk of relapse could likely be reduced by slowly tapering infusions. Indeed, the relatively quick tapering we adopted may have been responsible for some of the observed relapses. For example, three of the five patients (P10, P11, and P12) who had to switch to a new biotherapy showed a flare (Table 2) within 3 months after increasing treatment spacing. Saiki’s recent study showing the maintenance of a response rate greater than 90% in patients where treatment spacing was progressively increased initially to 5 weeks and then to 6 weeks also supports our contention [25]. Conversely, if TCZ is reduced directly from 4 to 6 weeks, the maintenance of a low activity level drops to about 60% [26]. We also found that plasma levels of TCZ, as well as ADAs, were not useful to predict flares after TCZ tapering.

## Conclusions

TCZ maintenance seems to be possible through progressive spacing of infusions in RA patients with sustained remission. Further studies should be conducted with larger number of patients to confirm this hypothesis, and to discover factors identifying patients that could benefit from this strategy.

## Data Availability

Please contact authors for data requests (Pers YM, M.D., Ph.D – email address: ympers2000@yahoo.fr).

## Abbreviations

ACPA: anti-citrullinated peptide antibodies
ACR: American College of Rheumatology
ADAb: antidrug antibodies
bDMARDs: biological Disease Modifying Anti-Rheumatic Drugs
cs DMARDs: conventional synthetic Disease Modifying Anti-Rheumatic Drugs
EULAR: European League Against Rheumatism
LDA: Low disease activity
MTX: methotrexate
RA: Rheumatoid arthritis
RF: Rheumatoid factor
RTI: Retreatment interval
TCZ: tocilizumab
TNF-α: tumor necrosis factor α
US: ultrasound
